# Fusion protein comprised of the two schistosomal antigens, Sm14 and Sm29, provides significant protection against *Schistosoma mansoni* in murine infection model

**DOI:** 10.1186/s12879-015-0906-z

**Published:** 2015-03-24

**Authors:** Shereen F Mossallam, Eglal I Amer, Radwa E Ewaisha, Amal M Khalil, Hamida M Aboushleib, Mohammed Bahey-El-Din

**Affiliations:** Department of Medical Parasitology, Faculty of Medicine, Alexandria University, Alexandria, Egypt; Department of Pharmaceutical Microbiology, Faculty of Pharmacy, Alexandria University, Alexandria, Egypt

**Keywords:** *Schistosoma mansoni*, Vaccine, Sm14, Sm29, Fusion protein, FSm14/29

## Abstract

**Background:**

*Schistosoma mansoni* infection represents a major cause of morbidity and mortality in many areas of the developing world. Effective vaccines against schistosomiasis are not available and disease management relies mainly on treatment with the anthelmintic drug praziquantel. Several promising schistosomal antigens have been evaluated for vaccine efficacy such as Sm14, Sm29 and tetraspanins. However, most investigators examine these promising antigens in animal models individually rather than in properly adjuvanted antigen combinations.

**Methods:**

In the present study, we made a recombinant fusion protein comprised of the promising schistosomal antigens Sm14 and Sm29. The fusion protein, FSm14/29, was administered to Swiss albino mice either unadjuvanted or adjuvanted with polyinosinic-polycytidylic acid adjuvant, poly(I:C). Mice were challenged with *S. mansoni* cercariae and different parasitological/immunological parameters were assessed seven weeks post-challenge. Data were analyzed using the ANOVA test with *post-hoc* Tukey-Kramer test.

**Results:**

Mice pre-immunized with unadjuvanted or poly(I:C)-adjuvanted fusion protein showed reduction of adult worm burden of 44.7 and 48.4%, respectively. In addition, significant reduction of tissue egg burdens was observed in mice immunized with the fusion protein when compared with the infected saline/adjuvant negative control groups and groups immunized with the individual Sm14 and Sm29 antigens. Light microscope and scanning electron microscope (SEM) investigation of adult worms recovered from FSm14/29-immunized mice revealed appreciable morphological damage and tegumental deformities. Histopathological examination of liver sections of immunized mice demonstrated reduced granulomatous and inflammatory reactions when compared with infected unvaccinated mice or mice immunized with the individual Sm14 and Sm29 antigens.

**Conclusion:**

The findings presented in this study highlight the importance of the fusion protein FSm14/29 as a potential vaccine candidate that is worthy of further investigation.

## Background

Schistosomiasis is considered to be one of the most serious parasitic infections in the world. It is estimated to infect more than 207 million in at least 74 countries worldwide [1]. The use of the anthelmintic drug praziquantel for treatment is the cornerstone of managing the disease in infected individuals. However, there are high re-infection rates in individuals who are inevitably exposed to the contaminated water [2].

There is currently no effective vaccine against schistosomiasis despite the innumerable efforts for its development in the last twenty-five years. Strong evidence supports the possibility of development of a vaccine against the different species of schistosomes [3,4]. Even non-sterilizing immunity against *Schistosoma* is expected to significantly reduce disease transmission and pathology because schistosomes do not replicate inside human hosts [5]. However, most investigators tested potential schistosomal vaccine antigens individually rather than in properly adjuvanted antigen combinations. It is unlikely that a single antigen would give the required protective results due to the complex structure of the different stages in the schistosomal life cycle besides the multiple immune responses involved.

Several promising antigens from *S. mansoni* can constitute the basis of a protective vaccine. The fatty-acid binding protein (FABP), Sm14, for instance, showed promising results in animal trials [6,7]. Sm14 is expressed in all life stages of the *S. mansoni* parasite [8]. In addition, due to structural similarity to a *Fasciola hepatica* antigen, Sm14 was tested as a potential vaccine against both schistosomiasis and fascioliasis [9,10]. Another important antigen is the tegumental protein Sm29 which is located on the surface of adult worms and schistosomula [11]. Interestingly, Sm29 is recognized preferentially by IgG1 and IgG3 from putatively resistant (PR) more than chronically infected (CI) individuals [11]. Putatively resistant individuals are characterized by being constantly exposed to infection but are negative for infection for over 5 years, having never been treated with an anthelmintic in addition to maintaining intense cellular and humoral immune responses to crude schistosomal antigenic preparations [12-15]. This, therefore, supports the usefulness of Sm29 as a potential vaccine candidate.

Investigators use different adjuvants in schistosomiasis vaccine research such as complete and incomplete Freund’s adjuvants, alum, CpG and polyinosinic-polycytidylic acid (poly (I:C)) [11,16,17]. Although the latter poly(I:C) adjuvant is a strong toll-like receptor 3 (TLR-3) agonist and is known to induce appreciable Th1 immune response [18], it has been less frequently used in schistosoma vaccine research than the former adjuvants.

We recently investigated the potential vaccine efficacy of Sm14 and Sm29 mixture for protection against schistosomiasis in murine model [19]. When the two antigens were combined, they could elicit good protection where 31.2% and 40.3% reduction of adult worm burden were attained in mice immunized with the unadjuvanted or poly(I:C)-adjuvanted combination, respectively. However, on a pharmaceutical scale, producing each antigen separately then mixing them together to get the final multi-antigen vaccine is a time- and money-consuming process. A more practical and economic approach is to create a fusion protein comprised of the desired antigens so that the vaccine fusion protein can be produced in a single biotechnology fermentation process. This clearly cuts down the cost of large scale production where the downstream purification procedure is carried out only once for the fusion protein rather than performing it twice, one for each antigen.

Consequently, in the current work, we examined the protective vaccine efficacy of a fusion protein comprised of the two promising schistosomal antigens Sm14 and Sm29. The fusion protein, FSm14/29, was investigated in Swiss albino mouse model both in an unadjuvanted form and in combination with the synthetic poly(I:C) adjuvant.

## Methods

### Vectors, culture media and incubation conditions

pQE31 vector (Qiagen GmbH, Hilden, Germany) was used for cloning of antigen genes. *Escherichia coli* TOP10 (Life Technologies Inc., New York, USA) and *E. coli* M15 (pREP4) (Qiagen GmbH, Hilden, Germany) were used as intermediate and expression hosts, respectively. Luria Bertani (LB) broth and agar were used for routine culture of *E. coli* strains. When required, ampicillin (Amp) and kanamycin (Km) were added to culture media at final concentrations of 100 and 25 μg/ml, respectively.

### Reverse-transcription and polymerase chain reaction (RT-PCR)

*S. mansoni* adult worms were obtained from the Schistosome Biological Supply Centre (SBSC), Theodor Bilharz Research Institute (TBRI), Giza, Egypt. Total RNA was extracted from the adult worms using Biozol® (Hangzhou Bioer Technology Co., Hangzhou, China) following manufacturer’s instructions. Briefly, adult worms were thoroughly cut using a scalpel, while immersed in 1 ml of Biozol® reagent. The homogenized worm suspension was subsequently vortexed in a sterile nuclease-free microcentrifuge tube with 0.3 g acid-washed glass beads (Sigma Aldrich, USA) for three one-minute beats with one-minute pauses on ice in between. This was followed by incubation on ice for 15 min and addition of 200 μL of chloroform. The microcentrifuge tube was vigorously shaken for 15 s, incubated for 15 min at room temperature and centrifuged. The upper aqueous layer containing total RNA was subsequently treated with DNase I then subjected to a final enrichment step to extract poly A^+^ mRNA by using the Oligotex® mRNA Mini Kit (Qiagen GmbH, Hilden, Germany) following manufacturer’s recommendations. In brief, total RNA was mixed with the Oligotex® resin where mRNA was captured from its poly A tail by the oligo-dT found on the resin. The resin was washed in a spin column and bound mRNA was subsequently eluted using the provided elution buffer.

Reverse transcription of mRNA was carried out at 42°C for 90 min using RevertAid™ H Minus First Strand cDNA Synthesis Kit (ThermoScientific Inc., Vilnius, Lithuania) where Oligo (dT)_18_ primer was used. PCR reactions were subsequently performed using high-fidelity *Pfu* DNA polymerase (ThermoScientific Inc., Vilnius, Lithuania) where cDNA was used as a template to amplify the *Sm14* and *Sm29* genes of *S. mansoni* [Genbank accession numbers XM_002580386 and AF029222, respectively]*.* Primers 5’- TAGTTTCTTGGGAAAGTGGAA-3’ and 5’-TTAGGATAGTCGTTTATAATTGC-3’ were used to amplify *Sm14* gene while primers 5’- GTGCGTTGCTACGTCTGTGATTATT-3’ and 5’-TTATTTTGTCATTCCGTTACATAGAT-3’ were used for *Sm29* amplification. For *Sm29*, we amplified the gene encoding amino acids 27–169 of the native Sm29 to exclude the N-terminal signal peptide and the C-terminal hydrophobic transmembrane domain [20].

### Creation of antigen gene fusion and cloning in *E. coli*

**Figure 1:**
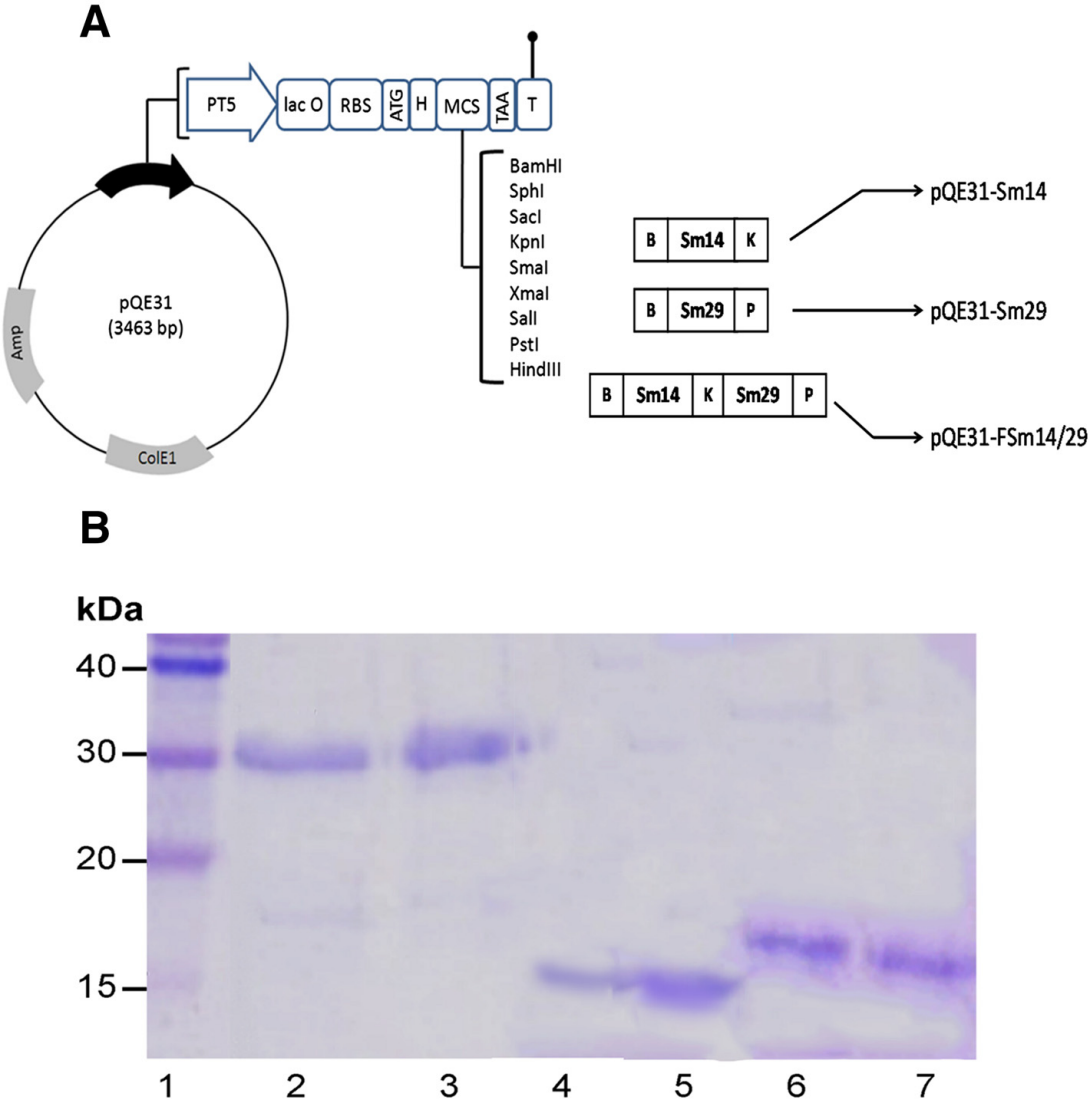
**Illustration of the created plasmids and purified antigens described in the study. (A)** Schematic representation of the construction of pQE31-FSm14/29, pQE31-Sm14 and pQE31-Sm29. Amp: β-lactamase coding sequence; ColE1: ColE1 origin of replication; PT5: T5 promoter; lac O: *lac* operator element; RBS: Ribosomal Binding Site; H: Hexahistidine tag; MCS: Multiple Cloning Site; T: Terminator; B: BamHI recognition site; K: KpnI recognition site; P: PstI recognition site. **(B)** SDS-PAGE of antigens purified from corresponding induced *E. coli* M15 (pREP4) strains. Lane 1, protein ladder; lanes 2 and 3, two successive elutions of the fusion antigen FSm14/29; lanes 4 and 5, two successive elutions of Sm14 antigen; lanes 6 and 7, two successive elutions of Sm29 antigen. Buffer E (pH 4.5) was used for elutions under denaturing conditions following induction with 0.3 mM IPTG at 25°C for 16 h.

To create a fusion protein comprised of Sm14 and Sm29, herein designated FSm14/29, the following procedure was carried out. The above obtained *Sm14* and *Sm29* amplicons were used as templates for the following two PCR reactions respectively. First, *Sm14* was amplified again using primers 5’- TGAAGGATCCGATGTCTAGTTTCTTGGGAAAGTGGAA-3’ and 5’- ACGCGGTACCGGATAGTCGTTTATAATTGC-3’ with BamHI and KpnI flanking restriction sites, respectively. Second, *Sm29* was amplified using primers 5’- TATAGGTACCGTGCGTTGCTACGTCTGTGATTATT-3’ and 5’-ACGCCTGCAGTTATTTTGTCATTCCGTTACATAGAT-3’ with flanking sites of KpnI and PstI, respectively. Both *Sm14* and *Sm29* amplicons were cut with KpnI then ligated together with T4 DNA ligase and the ligation reaction was used as a template to amplify the fusion gene using *Sm14* forward primer and *Sm29* reverse primer with BamHI and PstI recognition sites, respectively. The resulting fusion gene amplicon was cut with BamHI and PstI and was ligated to a similarly digested pQE31 plasmid. The resulting pQE31-(*FSm14/29*) was transformed into *E. coli* TOP10 as an intermediate cloning host. Plasmid pQE31-(*FSm14/29*) was subsequently extracted from *E. coli* TOP10 and transformed into the expression host *E. coli* M15 (pREP4) by electroporation where cells were plated onto Amp/Km-containing LB agar plates. Positive colonies were confirmed by colony PCR to ensure correct creation of the construct. Plasmids pQE31-*Sm14* and pQE31-*Sm29* containing the single antigen genes were also created and transformed into *E. coli* M15 (pREP4) using similar cloning procedures. Figure 1A illustrates the three plasmids created in this study.

### Expression and purification of *S. mansoni* antigens

Fresh cultures of engineered *E. coli* M15 (pREP4) strains were grown in LB broth containing Amp and Km at 37°C with vigorous shaking to an OD_600_ of 0.5. Protein expression was then induced using 0.3 mM isopropyl-β-D-thiogalactopyranoside (IPTG) for 16 h at 25°C. His-tagged proteins were purified by metal affinity chromatography from corresponding induced *E. coli* M15 (pREP4) strains under denaturing conditions using the procedure described by Qiagen (QIAexpressionist™). The bound protein was eluted twice (1 ml each) in buffer E (100 mM NaH_2_PO_4_, 10 mM Tris.Cl, 8 M urea, pH 4.5). Aliquots of elutions were checked by sodium dodecylsulfate-polyacrylamide gel electrophoresis (SDS-PAGE).

Western blot was also carried out using mouse anti-His tag antibody (Abgent Inc., San Diego, USA) as a primary antibody. Alkaline phosphatase-labeled anti-mouse IgG antibody (KPL Inc., Maryland, USA) was used as a secondary antibody and BCIP/NBT (KPL Inc., Maryland, USA) was used as a phosphatase substrate. Protein elutions were dialyzed against buffer containing descending urea concentrations (6, 4, 2 and 0 M urea) and were quantified by Bradford reagent. All purification and dialysis buffers were prepared in sterile endotoxin-free water. Absence of endotoxin in the final antigen preparation was ensured using the PYROTELL® Limulus Amebocyte Lysate (LAL) test (Associates of CAPE COD Inc., Massachusetts, USA) with a sensitivity level (λ) of 0.25 EU/ml (Endotoxin unit/ml).

### Animal immunization protocol

Female Swiss albino mice, 6–8 weeks old, were divided into eight groups, ten mice each. The antigens FSm14/29, Sm14 and Sm29 were administered, in the presence/absence of poly(I:C) adjuvant (Invivogen, Toulouse, France), intraperitoneally (IP) to mice in corresponding groups on days 1, 14 and 28. FSm14/29, Sm14 and Sm29 were given at doses of 20, 20 and 10 μg/mouse respectively while the poly(I:C) adjuvant was given at a dose of 50 μg/mouse. These concentrations were chosen based on previously published data for Sm14 and Sm29 [21,22]. Two negative control groups injected with sterile saline or poly(I:C) adjuvant were also included. All antigen doses were prepared in sterile endotoxin-free saline. Two weeks after the last booster, serum samples were collected and mice were challenged with *S. mansoni* cercariae. Each mouse was infected with 100 cercariae as previously described [23,24]. Animals were euthanized seven weeks postinfection and different parasitological parameters were evaluated. Two independent vaccine trials were carried out. All animal experiments and procedures were approved by the animal ethics committee of Alexandria University (licence no. 04085) based on national regulations for animal experimentation.

### Determination of antigen-specific serum IgG1 and IgG2a using enzyme-linked immunosorbent assay (ELISA)

Antigen-specific immunoglobulins IgG1 and IgG2a were determined in serum samples from mice two weeks after the last vaccine booster as previously described [25] with slight modification. Briefly, an ELISA assay was developed where ELISA plates (Greiner Bio-One, Frickenhausen, Germany) were coated with 100 μl of individual antigens at a final concentration of 10 μg/ml. Serum dilutions were added to the plates after blocking with 5% skimmed milk in phosphate-buffered saline (PBS). A His-tagged irrelevant antigen, listeriolysin O [26], was used as a negative control in the ELISA test in order to exclude any probable reactivity against the His-tag itself. Plates were subsequently washed and peroxidase-labeled goat anti-mouse IgG1 or anti-mouse IgG2a antibodies (Abcam, Massachusetts, USA) were added for 1 h. Following extensive washing, the substrate 3,3’,5,5’-tetramethylbenzidine (TMB) was added to the wells followed by 1 M sulfuric acid to stop the reaction. Absorbance readings were measured at 450 nm in a microplate reader (Biotek, Winooski, USA).

### Evaluation of parasitological parameters in challenged mice

The following parasitological parameters were assessed in euthanized animals.

i) Estimation of the adult worm load and microscopical examination
  Hepatic and porto-mesenteric vessels were perfused and adult worms were recovered and counted [24]. The protection level was evaluated based on the percentage reduction (R) as follows: R = [(C-V)/C] X100, where C is the mean worm count in the negative control group and V is the mean worm count in vaccinated groups [23,24]. Recovered worms were processed as previously described [27,28] for carmine staining and for examination by scanning electron microscopy (SEM).
ii) Examination of tissue egg loads
  Livers and intestines of both the vaccinated and control animal groups were isolated and weighed. Eggs were counted per gram tissue according to the method described by Cheever [29].
iii) Histopathological examination of livers
  Livers of mice from all studied groups were subjected to histopathological examination following hematoxylin and eosin (H&E) and Masson’s Trichrome (MT) staining [30]. Pathological changes in the hepatic parenchyma were examined and the granulomata mean number and diameters were determined [31].

### Statistical analysis

Data were statistically analyzed with the ANOVA test followed by *post-hoc* Tukey-Kramer test using GraphPad InStat version 3.1 software. Results with *p* value < 0.05 were considered statistically significant.

## Results

### Recombinant expression and detection of antigens

The DNA sequence of all created constructs was determined (GATC Biotech, Cologne, Germany) and was found to be identical to the database of target accession numbers through sequence alignment. Proper antigen production by induced *E. coli* M15 (pREP4) strains was confirmed by SDS-PAGE (Figure 1B) and Western blot (data not shown).

### Antigen-specific serum antibodies

**Figure 2:**
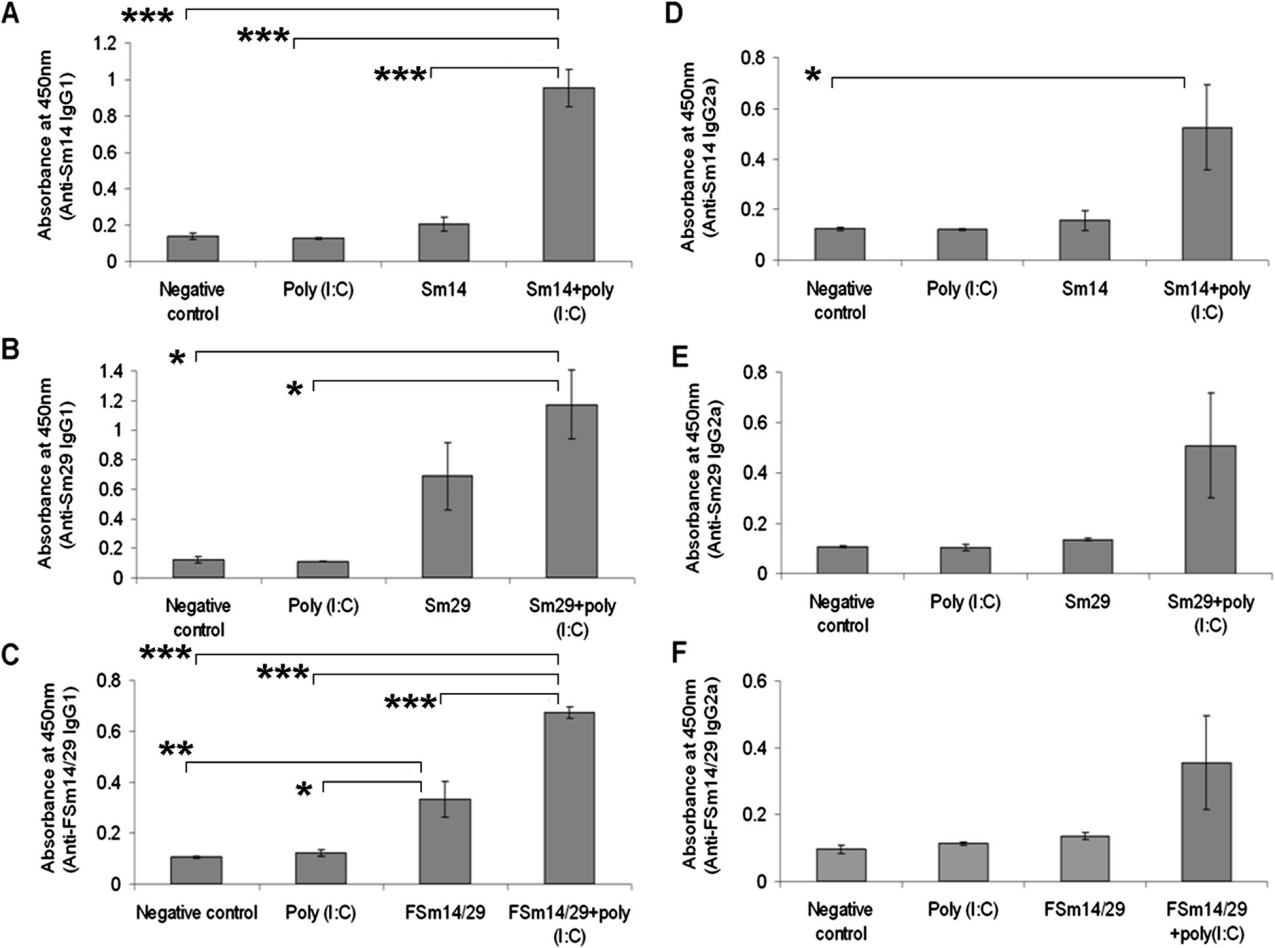
**Antigen-specific IgG1 and IgG2a antibodies in sera of immunized mice.** ELISA plates were coated with corresponding antigens and serum samples were tested for the presence of anti-antigen IgG1 **(A, B and C)** and anti-antigen IgG2a **(D, E and F)**. The shown results are at 1/250 serum dilution where error bars represent the mean reading +/− SE (standard error of the mean). (*) p < 0.05; (**) p < 0.01; (***) p < 0.001 by the ANOVA test and *post-hoc* Tukey-Kramer test. Asterisks are shown only for groups that showed significant statistical difference.

Serum samples were collected from immunized mice two weeks after the last booster for specific antibody detection by ELISA. The single antigens Sm14 and Sm29 elicited specific IgG1 antibodies in immunized mice mainly in groups vaccinated with corresponding adjuvanted antigens when compared with the negative control and poly(I:C) groups (Figure 2A and B). The fusion antigen FSm14/29 stimulated significant specific IgG1 in animals immunized with unadjuvanted or poly(I:C)-adjuvanted fusion antigen when compared with the negative control and poly(I:C) groups (Figure 2C). Upon comparing the results of IgG1 against FSm14/29 and the individual antigens, it was found that anti-Sm14 IgG1 antibodies (mean absorbance 0.95) was significantly higher (p = 0.03) than anti-FSm14/29 IgG1 (mean absorbance 0.67) in groups immunized with the poly(I:C)-adjuvanted corresponding antigens (Figures 2A and C). On the other hand, specific IgG2a antibody levels were not statistically significant in all mice groups except for the poly(I:C)-adjuvanted Sm14 vaccinated group when compared to the saline-treated negative control group (Figure 2D-F). The mean absorbance values for IgG2a ELISA detection were generally higher in mice immunized with poly(I:C)-adjuvanted antigens than in mice immunized with unadjuvanted antigens, but difference was not statistically significant (Figure 2D-F).

### Protection against *S. mansoni* infection in mice immunized with the fusion antigen

Groups immunized with FSm14/29 or the poly(I:C)-adjuvanted FSm14/29 showed significant reduction of adult worm burden of 44.7 and 48.4% respectively (Table 1). On the other hand, total adult worm burden was not significantly reduced in mice immunized with the single antigens when compared with the negative control group. As regards the liver egg burden, the most significant reductions, 82.8 and 73.5%, were achieved in mice immunized with poly(I:C)-adjuvanted and unadjuvanted fusion protein, respectively (Table 1). Similarly, the intestinal egg count was minimal in mouse groups immunized with the fusion protein where burden reduction reached 72.8 and 76.6% for the adjuvanted and unadjuvanted FSm14/29 antigen, respectively (Table 1). All percentage reduction values were calculated relative to the infected saline-injected (unimmunized) negative control group. Importantly, the adult worm burden and tissue egg loads were statistically lower in groups immunized with the adjuvanted or unadjuvanted FSm14/29 (p < 0.05) when compared with all other groups including groups immunized with the individual antigens, the saline negative control and the adjuvant groups. However, there was no statistically significant difference between the groups immunized with either the poly(I:C)-adjuvanted FSm14/29 or the unadjuvanted FSm14/29 concerning the reduction of adult worm and tissue egg burdens.

**Table 1:** **Assessment of different parasitological parameters and protective efficacy in pre-immunized mice challenged with** ***S. mansoni*** **cercariae**

**Animal group**	**Adult worm burden ± SE**	**%R**	**Eggs/gram liver ± SE**	**%R**	**Eggs/gram intestine ± SE**	**%R**	**Mean no. of granulomas/LPF ± SE**	**%R**	**Mean granuloma diameter (μm) ± SE**	**%R**
**Negative control**	22.714 ± 2.6	-	2489.857 ± 172	-	3803.571 ± 352.4	-	10.714 ± 0.5	-	285.857 ± 12.1	-
**Poly (I:C)**	21.778 ± 2.4	4.1^NS^	1918.556 ± 220	22.9^NS^	3586.444 ± 181.6	5.7^NS^	8.444 ± 0.5	21.2^†^	254.444 ± 10.1	11^NS^
**Sm14**	22.1 ± 1.9	2.7^NS^	2452.2 ± 167.2	1.5^NS^	3806.1 ± 195.5	0.1^NS^	7.8 ± 0.29	27.2**	216.778 ± 6.8	25**
**Sm14+ poly (I:C)**	19.429 ± 3.1	14.5^NS^	1878.571 ± 104	24.6^NS^	2702 ± 158.6	28.96^†^	8 ± 0.58	25.33*	227.857 ± 11.3	20.3*
**Sm29**	18.625 ± 2	18^NS^	1489.5 ± 97.1	40.2*	2604.875 ± 132.1	31.5*	7.375 ± 0.46	31.2**	219.375 ± 7.9	23.3**
**Sm29+ poly (I:C)**	21.222 ± 1.5	6.6^NS^	2042.111 ± 230	18^NS^	3502.889 ± 219	7.91^NS^	7.556 ± 0.5	29.5**	240 ± 10.1	16^†^
**FSm14/29**	12.556 ± 1.2	44.7^†^	659.222 ± 116.5	73.5**	888.667 ± 89.3	76.64**	4.1 ± 0.23	61.7**	152.556 ± 7.7	46.6**
**FSm14/29 + poly(I:C)**	11.714 ± 1.7	48.4^†^	428.857 ± 103.6	82.8**	1033.857 ± 128.4	72.82**	3 ± 0.31	72**	102.571 ± 6.4	64.1**

(^NS^): statistically non-significant; (^†^): *p* value < 0.05; (*): *p* value < 0.01; (**): *p* value < 0.001 when compared to the negative control group using the ANOVA test with *post-hoc* Tukey-Kramer test. SE: standard error of the mean. LPF: low power field. %R: percentage reduction as compared to the negative control. All results shown for groups immunized with adjuvanted or unadjuvanted FSm14/29 were significantly different (*p* < 0.05) from all other groups including the negative control, the adjuvant and the individual antigens groups using the ANOVA test with *post-hoc* Tukey-Kramer test.

### Alterations in adult worms detected by light microscopy

**Figure 3:**
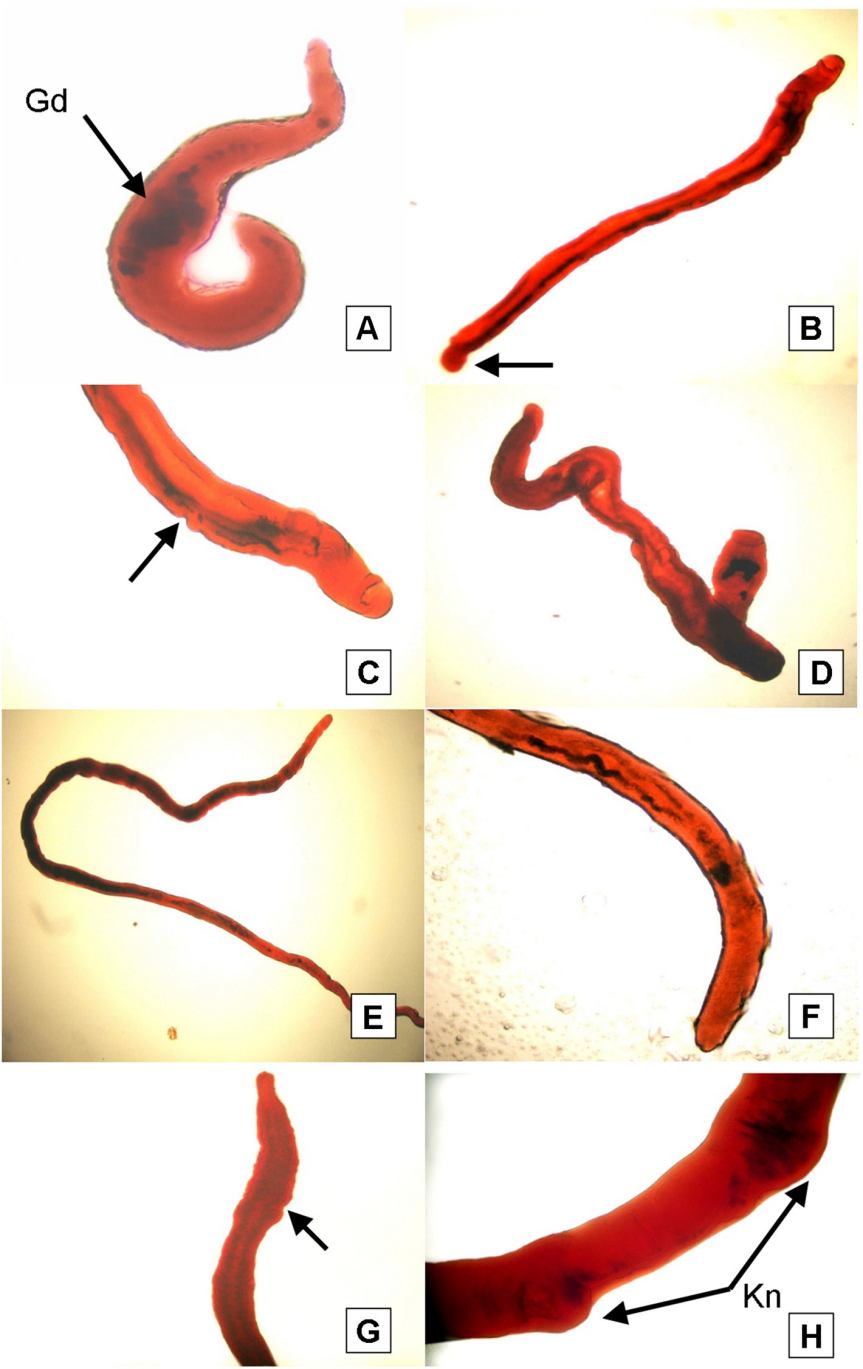
**Light microscopic appearance of carmine-stained adult worms recovered from FSm14/29-immunized mice. (A)** Abnormally shortened adult male worm exhibiting multiple gut dilatations (Gd). The testes were not clearly seen due to dilatations of the gut in the anterior part of the worm. **(B)** Slightly elongated male with bulging (arrow) in the posterior end. **(C&D)** Male worm demonstrating gut dilatation which hides the appearance of the testes, with ill-defined oedematous suckers, and remarkable tegumental irregularities in the worm outline. **(E)** An example of an elongated female. **(F&G)** Posterior ends of females possessing universal pronounced tegumental oedematous irregularities (arrows) or even wrinkled posterior ends. **(H)** Occasional swollen knots (Kn) in a female mid-body. Sparseness of the pigment content of the gut was noticed in all examined female worms with indistinct vitelline gland lobulations and ill-defined ovarian boundaries.

Adult worms obtained from mice immunized with the unadjuvanted or poly(I:C)-adjuvanted FSm14/29 showed noticeable deformities. These include tegumental irregularities and occasional swollen knots as shown in details in Figure 3 which shows representative pictures for worms recovered from the infected FSm14/29-immunized group. None of these observed deformities were detectable in adult worms recovered from all other experimental groups including the infected unvaccinated negative control group, the adjuvant group and groups immunized with the individual antigens. In addition, we found that both male and female adult worms recovered from mice previously immunized with adjuvanted or unadjuvanted FSm14/29 showed statistically significant reduction (p <0.05) in their perimeters when compared with the negative control group (data not shown).

### Alterations in adult worms as determined by scanning electron microscopy (SEM)

Since FSm14/29, whether adjuvanted or unadjuvanted, showed the best protection results against *S. mansoni* infection (Table 1), we decided to further examine the ultrastructural changes of the adult worms recovered from groups immunized with FSm14/29 using SEM.

**Figure 4:**
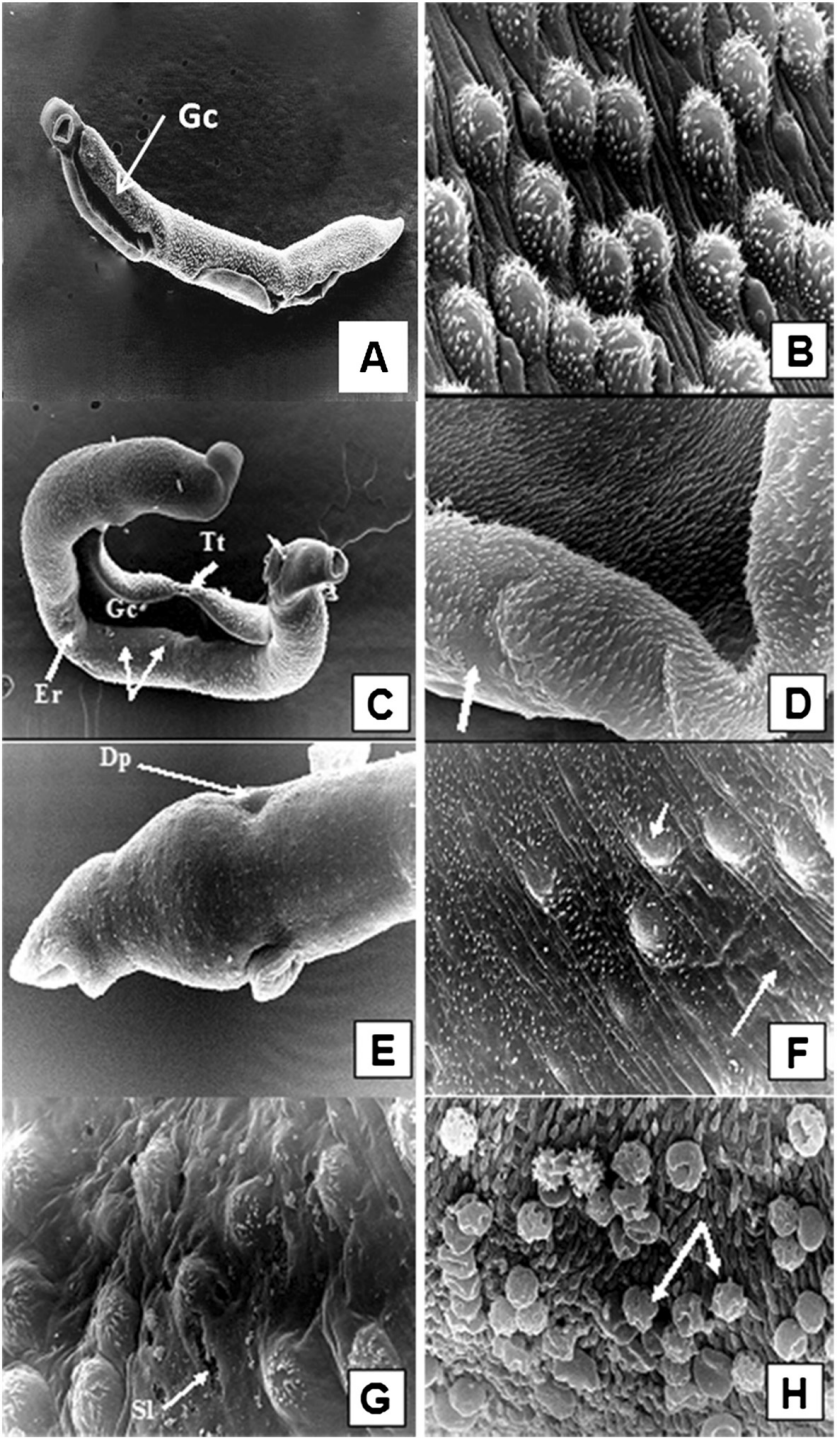
**Scanning electron micrographs of adult** ***S. mansoni*** **male worms recovered from unimmunized group (A and B) and from FSm14/29-immunized group (C-H). (A)** A male worm obtained from infected control group showing normal body configuration, typical oral and ventral suckers, and distinct tegumental structure with evident normal spines on the dorsal surface behind the beginning of the gynecophoric canal (Gc), X200. **(B)** Tegument of the middle dorsolateral region showing numerous large tubercles, each bearing numerous spines. Intertubercular ridges and ciliated sensory papillae are obvious, X 5,000. **(C)** Tegumental surface alterations in adult male from infected FSm14/29-immunized group demonstrating widening of the gynecophoric canal (Gc), loss of some areas of the tegumental tubercles and ridges (arrows), surface erosions (Er), tegumental tear (Tt), and bossing of the terminal end of the worm, X200. The posterior end shows several areas of bulging. **(D)** Higher magnification of a conspicuously widened gynecophoric canal, along with restricted spine loss (arrow), X5,000. **(E)** Swollen area between the oral and ventral suckers showing circumferentially arranged ridges with sensory papillae rows. An evident dorsal dimple is witnessed (Dp), along with suckers oedema and blebbing, X2,000. **(F)** Eminent flattening of the dorsal tubercles along with shortened spines which were irregularly positioned or even lost in some areas (arrows), X5,000. **(G)** Altered dorsal tubercles revealed peeling and focal sloughing (Sl) of their tegument. Tubercular and inter-tubercular coarse bumpy tegumental irregularities were observed, with occasional burst of some blistered tubercles, X5,000. **(H)** Host leucocytic (arrows) attachment to the worm surface with evident spine blunting, X7500.

Male adult worms obtained from unimmunized control mice showed normal configuration and tegumental structure, with evident normal spines on the dorsal surface behind the beginning of the gynecophoric canal (Figure 4A). The dorsolateral surface of the mid-body was covered by tubercles of uniform size and distribution, covered by typical spines and interspaced with tegumental ridges and ciliated sensory papillae (Figure 4B).

On the contrary, SEM examination of male worms recovered from mice immunized with unadjuvanted or poly(I:C)-adjuvanted FSm14/29 showed marked extensive tegumental alterations. These appeared in the form of conspicuous widening of the gynecophoric canal in a considerable number of the examined worms (Figure 4C and D). Swelling and small sized blebbings in the lips of the suckers were also present (Figure 4E). Worms exhibited eminent flattening of their dorsal tubercles along with shortened spines which were irregularly positioned or even lost in some areas (Figure 4F). Furthermore, altered dorsal tubercles revealed peeling and focal sloughing of their tegument, in association with tubercular and inter-tubercular coarse bumpy tegumental irregularities, with occasional burst of some blistered tubercles (Figure 4G). Host leucocytic attachment to the worm surface was a commonly witnessed feature (Figure 4H).

**Figure 5:**
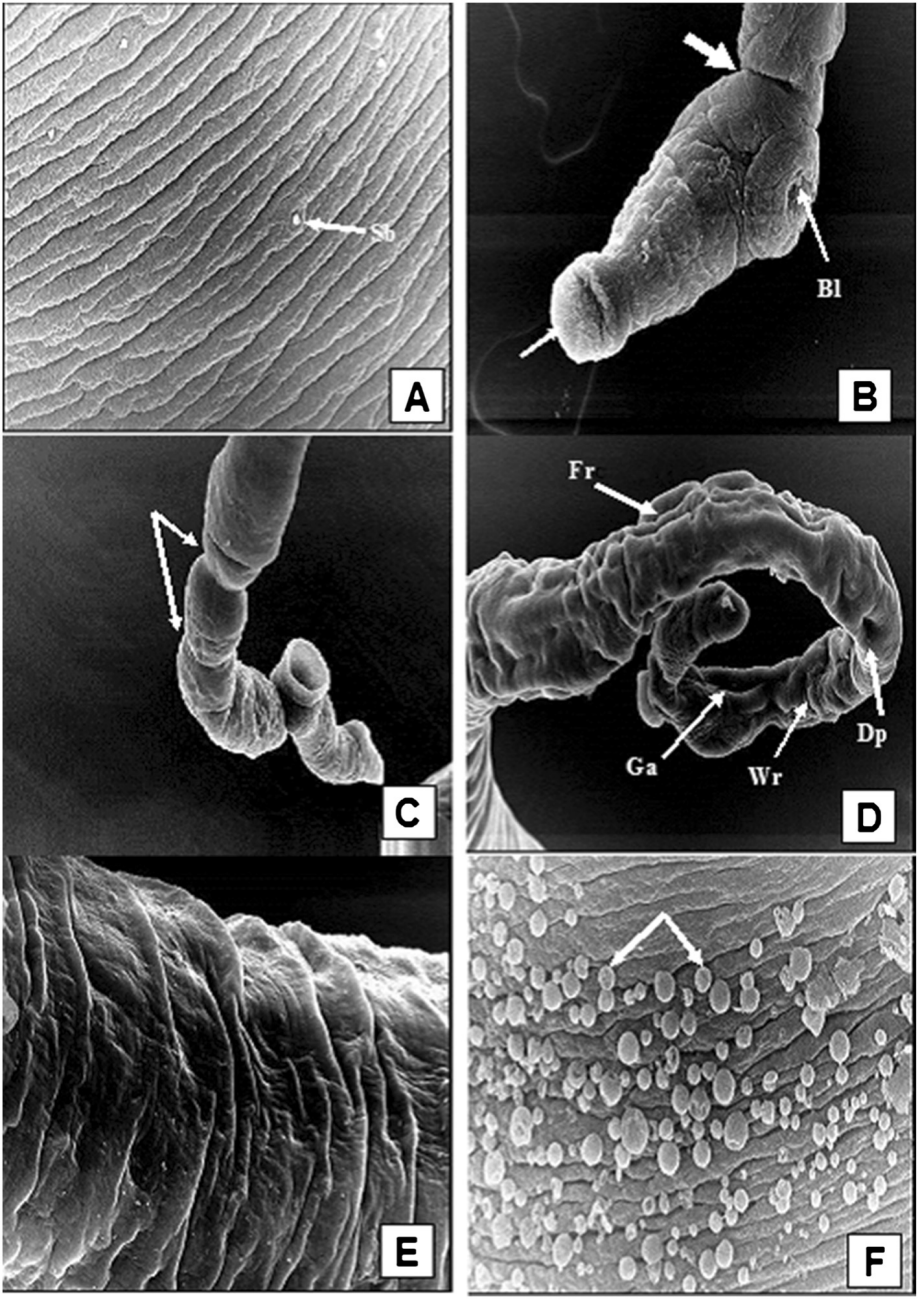
**Scanning electron micrographs of adult** ***S. mansoni*** **female worms recovered from unimmunized group (A) and from FSm14/29-immunized group (B-F). (A)** The dorsolateral surface of a female recovered from the unimmunized control group showing fine circular ridges interspaced with regular clefts and carrying conspicuous sensory bulbs (Sb), X3,500. **(B)** The anterior end of a female worm recovered from FSm14/29-immunized animals exhibiting extensive swelling of the forebody tegument, with evident strangulation (thick arrow), associated with small sized blebbings (Bl) in the lips of their suckers. Spines lining the edematous suckers were short, irregularly positioned and chubby in appearance (thin arrow), X500. **(C)** A convoluted female expressing irregularly positioned constrictions in various areas of its body (arrows), besides loss of the mid-body circular ridges with complete destruction of the external surface of the forebody, X750. **(D)** Kinky female surface swellings alternating with dimples (Dp), furrows (Fr), gappings (Ga), and wrinkling (Wr), X750. **(E)** Higher magnification verifying that the linear transverse tegumental ridges encircling the body worm were replaced by swelling induced loosing of the clefts, and fusions of ridges. This resulted in the formation of long irregular and disorganized splits, X3,500. **(F)** Host leucocytes attached to the tegument of the female surfaces (arrows), X7,500.

Figure 5 shows representative SEM images of female worms obtained from control unimmunized mice and from mice immunized with the fusion protein. The tegumental surface of female worms isolated from unimmunized mice was generally smooth with fine circular ridges interspaced with regular clefts and was carrying conspicuous sensory bulbs throughout the dorsal surface (Figure 5A). On the other hand, adult female worms obtained from FSm14/29-immunized mice exhibited drastic alterations in the form of extensive swelling of the forebody tegument associated with edema and small-sized blebbings in the lips of their suckers. Spines lining the suckers were short, irregularly positioned and chubby in appearance (Figure 5B). Furthermore, females were convoluted expressing irregularly positioned constrictions in various areas of their bodies (Figure 5C). Their surfaces showed swellings alternating with dimples and depressions, gappings, and wrinkling (Figure 5D). Higher magnification verified that the linear transverse tegumental ridges encircling the worm body were not observable, being replaced by swelling induced loosing of the clefts, and fusions of ridges. This resulted in the formation of long irregular and disorganized splits (Figure 5E). Similar to altered males, it was highly evident that host leucocytes were attached to the body of the female worms in various areas (Figure 5F).

### Histopathological changes

All immunized groups showed significant reduction in both number and size of liver granulomata when compared with the negative control group (Table 1). Groups immunized with the unadjuvanted or poly(I:C)-adjuvanted fusion protein showed the most significant reduction in mean granuloma number per low power field of 61.7 and 72% respectively when calculated with respect to the saline negative control group. In addition, percentage reduction of mean granuloma diameter of these two previous groups was 46.6 and 64.1% respectively when calculated with respect to the saline negative control group. It is important to note that the granuloma number and diameters in the groups immunized with adjuvanted or unadjuvanted FSm14/29 were statistically lower (p <0.001) than all other groups immunized with the single antigens, the saline negative control group and the adjuvant group. It is also noteworthy that reduction in the mean granuloma diameter was statistically significant (*p* < 0.01) in mice immunized with poly(I:C)-adjuvanted FSm14/29 when compared with those immunized with the unadjuvanted fusion protein.

**Figure 6:**
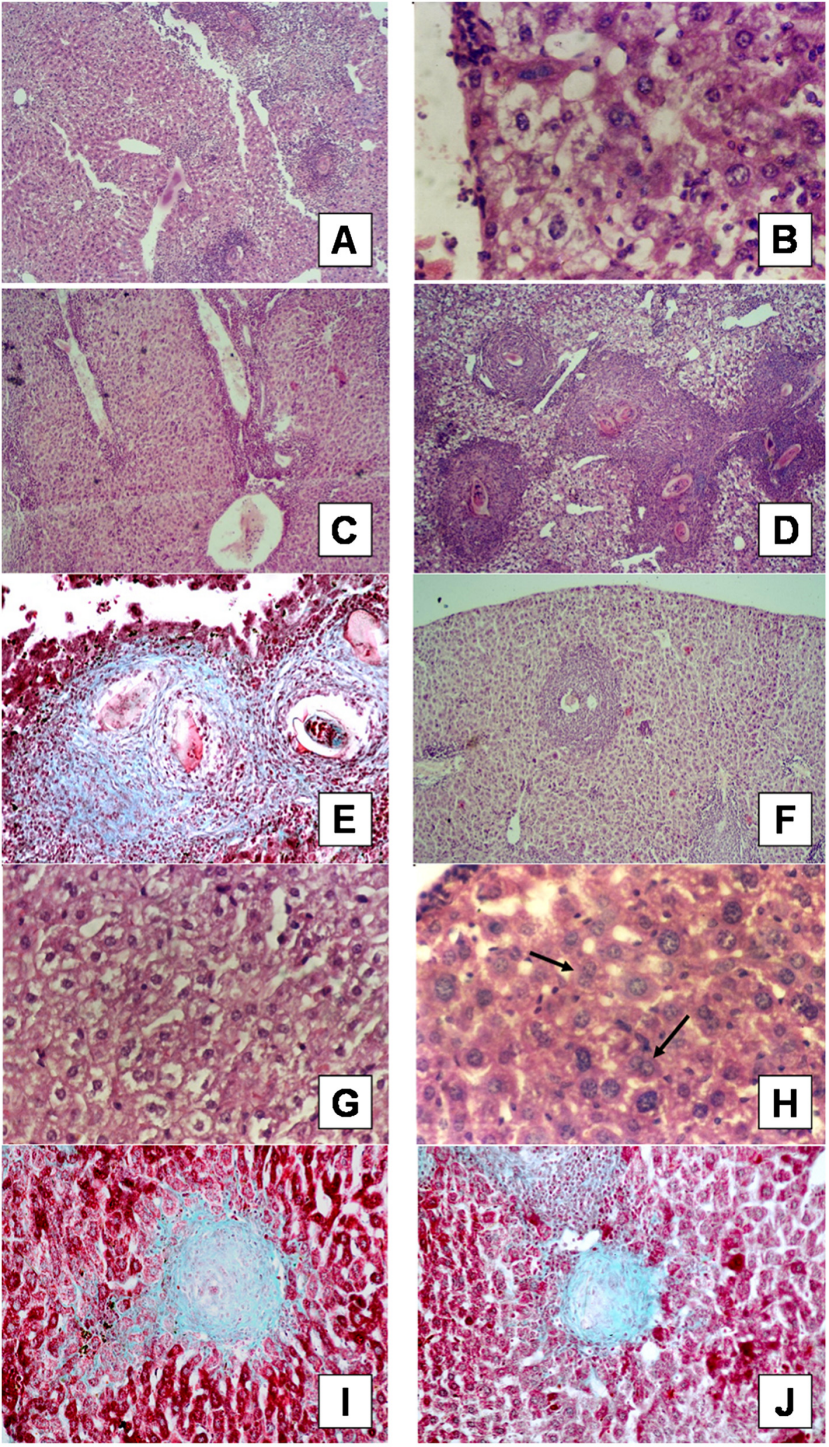
**Liver histological sections form infected unimmunized control mice (A-E) and FSm14/29-immunized mice (F-J). (A)** Liver section of infected unimmunized mouse showing Kupffer cells’ hyperplasia (H&E x100). **(B)** Liver section of an infected mouse showing hydropic degeneration and ballooning of hepatic cells (H&E x400). **(C)** Liver section of an infected mouse showing lymphocytic infiltration of portal tract with marked dilatation and proliferation of blood vessels (H&E x100). **(D)** Liver section of an infected mouse showing numerous large size cellular granulomata (H&E x100). **(E)** Liver section of an infected mouse showing non-degenerated well developed *S. mansoni* egg trapped within cellular granuloma (MT x400). **(F)** Liver section of infected FSm14/29-immunized mouse showing decreased number and size of cellular granulomata surrounding degenerated eggs (H&E x100). **(G)** Liver section of infected FSm14/29-immunized mouse showing lesser degree of cellular swelling and inflammatory infiltration (H&E x400). **(H)** Liver section of infected FSm14/29-immunized mouse showing regenerative activity represented by hepatocyte binucleation (arrows) (H&E x400). **(I & J)** Liver sections of infected FSm14/29-immunized mouse showing small well-circumscribed fibrous granulomata with degenerated eggs (MT X400).

Liver sections of unimmunized infected control group showed typical histopathological features as shown in Figure 6A-E. Abundant large irregular cellular granulomata, in which *S. mansoni* eggs were surrounded mainly by epithelioid cells and infiltrating lymphocytes were observed with no sign of formation of collagen fibers. In groups immunized with unadjuvanted or poly(I:C)-adjuvanted FSm14/29, fewer and smaller cellular granulomata were seen and parenchymal changes were milder than controls (Figure 6F-J). Degenerated eggs were surrounded mostly by numerous eosinophils, neutrophils, lymphocytes and few epithelioid cells with evident fibroblastic proliferation (Figure 6 I and J).

## Discussion

We recently reported the beneficial effects of combining Sm14 and Sm29 antigens for vaccination against *S. mansoni* [19]. The mixture of those two antigens adjuvanted with poly (I:C) could produce significant reduction in adult worm burden (40.3%), liver egg burden (68.2%) and intestinal egg burden (57.9%) in immunized mice [19]. In the current study, we tested a fusion protein comprised of Sm14 and Sm29 for potential vaccine protective efficacy against schistosomiasis. The use of FSm14/29 is a more cost-effective approach upon large scale pharmaceutical production of the vaccine. A similar approach is used in the commercial production of insulin by *E. coli* where the proinsulin molecule, that contains the two peptide chains of insulin, is produced at large scale. This latter method is widely used nowadays instead of the old method that involved producing the two insulin peptide chains separately in *E. coli* then combining them [32].

Investigational vaccines against *S. mansoni* are more likely to succeed if they can elicit robust immune responses against multiple antigenic targets in the schistosomes. Animals immunized with either unadjuvanted or poly(I:C)-adjuvanted FSm14/29 showed 44.7 and 48.4% reduction of adult worm burden, respectively. Furthermore, significant reduction of tissue egg burden and granulomata was also achieved. The protective results shown in this study for FSm14/29, whether adjuvanted or not, were slightly higher than what we previously reported for the combination of Sm14 and Sm29 [19]. This demonstrates the potential value of FSm14/29 fusion protein as a cost-effective and efficacious vaccine candidate when compared with the combination of Sm14 and Sm29.

Under the experimental conditions of our study, poly(I:C)-adjuvanted or unadjuvanted individual Sm14 and Sm29 antigens did not produce statistically significant reduction in adult worm burden when compared with the negative control group (Table 1). Upon reviewing the literature, we found that different experimental conditions, different adjuvants and variable vaccine protective results were reported for Sm14 and Sm29. As regards Sm14, vaccine protection results ranged from lack of protection to variable degrees of protection. When mice were immunized with Sm14 adjuvanted with alum hydroxide, no reduction in adult worm burden was observed by Fonseca and colleagues although high levels of antigen-specific IgG1 were observed [33]. In addition, oral immunization with *Salmonella* harboring an Sm14-based DNA vaccine did not result in any reduction of adult worm burden in immunized mice [34]. Furthermore, intranasal administration of Sm14 or Sm14 fused to cholera toxin subunit B (CTB) failed to produce significant protection against *S. mansoni* infection in BALB/c mice [35]. Nevertheless, different protection levels were reported in animals immunized with Sm14 ranging from 25% to 67% [10,22,33]. Concerning the schistosomal antigen Sm29, 51% reduction in adult worm burden was observed in C57BL/6 mice immunized with Sm29 adjuvanted with complete and incomplete Freund’s adjuvants [11]. When C57BL/6 mice were immunized with a mixture of tegumental proteins composed mainly of Sm200 and Sm29, only 42% reduction of adult worm burden was achieved [36]. More recently, C57BL/6 mice immunized with Sm29 plus CpG/alum mixture showed only 20.36% reduction of adult worm burden [17]. Moreover, in the same study, immunization with two chimeric vaccine candidates composed of the two schistosomal antigens Sm29 and SmTSP-2 resulted in protection levels ranging from 27.84 to 34.83% [17]. This apparent discrepancy seen in the literature can be attributed to the different experimental conditions used by different research groups. Indeed, different adjuvants, routes of administration, antigen doses, vaccination regimens and even mouse strains were used by different investigators. This renders the direct comparison between published data unfeasible and results should be assessed based on the pertinent experimental conditions.

In the present study, we tested the fusion protein FSm14/29 in the presence and absence of poly(I:C) adjuvant. Poly(I:C) is reported to induce T helper type 1 (Th1) immune response with high levels of IFN-γ and IgG2a [16,37]. Th1 immune response is reported to be protective against murine schistosomiasis [33]. Both IgG1 and IgG2a antibodies were observed against FSm14/29 especially in mice immunized with the poly(I:C)-adjuvanted fusion protein. However, anti-FSm14/29 IgG2a levels were not statistically significant (Figure 2F). It was previously reported that IgG1 and IL-10-mediated immune responses downregulate the granulomatous hypersensitivity reaction of schistosomiasis [38]. This fits well with the reduction of granulomata size and number observed in mice immunized with unadjuvanted or poly(I:C)-adjuvanted FSm14/29. Indeed, high levels of specific IgG1 antibodies against Sm14 and Sm29 were detected in the sera of individuals resistant to schistosomiasis in endemic areas [20,39]. Although poly(I:C) is known to induce Th1-biased immune response, Zhou and coworkers found that poly(I:C) can induce a T helper type 2 (Th2) response in non-obese diabetic mice upon long-term administration [40]. Commercial poly(I:C) adjuvant has different molecular weights ranging from 0.2 to 8.5 kb depending on the supplier. In our study, we used high molecular weight (HMW) poly(I:C) (1.5-8 kb) purchased from Invivogen. It was reported that the molecular weight of poly(I:C) has a significant impact on its immunomodulatory actions leading sometimes to opposite effects on immune cells [41]. In addition, some investigators use the more stable poly ICLC, which is poly(I:C) stabilized with poly-lysine and carboxymethylcellulose, in their experiments in order to obtain robust results [42]. All these factors are under investigation in order to determine the optimum conditions required to maximize vaccine protective efficacy. Determination of detailed cytokine profile following vaccination would certainly help in choosing the most suitable grade of poly(I:C). Doubling the poly(I:C) adjuvant dose from 50 to100 μg per mouse is another option which might enhance its Th1 effects. In fact, the manufacturer of poly(I:C) used in our experiments (Invivogen; www.invivogen.com) recommends a dose range of 10–100 μg of adjuvant per mouse.

It is also noteworthy that there were significant levels of IgG1 against Sm14 and Sm29 besides FSm14/29 (Figure 2A-C). Nevertheless, protection was pronounced only in groups immunized with FSm14/29. This finding indicates that IgG1 role in protection against schistosomiasis is only partial and Th1 cell-mediated immune response might be required [33].

In the present study, there has been interestingly a significant reduction in the liver and intestinal egg burden in groups immunized with the unadjuvanted or poly(I:C)-adjuvanted FSm14/29 (Table 1). These results may indicate either an anti-fecundity effect of the vaccine or merely a delayed inception of egg laying [43]. Generation of developmental oograms and examination of egg laying at different experimental intervals should be performed to clarify this point [43,44].

Morphological alterations of adult worms give an indication of strong immune responses elicited by tested vaccines. The morphological changes noticed here are probably because the two antigens used in this study are easily accessed by the host immune system. Sm14 is a FABP localized in tissues near the interfaces of host/parasite contact including the basal lamella of the tegument [8], while Sm29 is a tegumental protein located on the surface of adult worms and schistosomula [11]. Immunization with the fusion protein FSm14/29, which is composed of a crucial couple of antigens, primed the immune system against the challenge infection resulting in tegumental changes as verified by the SEM study. Abdeen and coworkers immunized rabbits with irradiated *S. mansoni* cercariae and used the rabbits’ serum to passively immunize mice infected with *S. mansoni*. They observed similar tegumental damage and ultrastructural changes in *S. mansoni* adult worms recovered from infected mice [45].

In our study, scanned convoluted adult worms exhibited drastic tegumental and sucker alteration, with noticeable host leucocytic attachment to their surfaces. Conspicuous widening of gynecophoric canals of some males has probably hindered their appropriate coupling with females. It is well reported that muscular action of the clasping male helps females to get blood nutrition from the host [46]. In addition, decoupling has other negative effects on females because males are responsible for physically transporting the females and fertilizing oocytes [47].

Breaking and erosion of tubercles and ridges of adult worms recovered from FSm14/29-immunized mice were evident in our study. This exposed the basal lamina and eventually resulted in focal sloughing of the tegument. Such drastic tegumental changes practically expose more underlying antigenic determinants to the host immune recognition and effector functions culminating in enhanced vaccine protective efficacy.

Since most chronic morbidity features of schistosomiasis are related to the egg formation rather than the adult worms, minimizing tissue egg loads and granuloma formation would be a desirable goal of any successful vaccine against *S. mansoni.* The fusion protein FSm14/29, whether unadjuvanted or poly(I:C)-adjuvanted, showed significant reduction in both granulomata number and size in challenged immunized mice. Diminished granuloma size may suggest an effect on the immune response around the parasite ova in the tissues. Moreover, the granulomata showed proliferation of fibrocytes and increase in the amount of fibrous tissue that was obviously seen in Masson’s trichrome (MT)-stained sections suggesting that there is an attempt of tissue healing.

## Conclusion

Overall, we have demonstrated that vaccination with the fusion protein FSm14/29 offers significant protection against *S. mansoni* infection in Swiss albino mice. Immunization resulted in significant reduction of adult worm burden, tissue egg loads and liver granuloma formation. Deleterious structural changes were also observed in adult worms recovered from immunized mice. Optimization of experimental conditions and the adjuvant used should greatly improve the protection outcome and consolidate the concept of using multi-antigen fusion proteins as vaccine candidates against *S. mansoni.*

## References

[1] King CH (2009). Toward the elimination of schistosomiasis. N Engl J Med.

[2] Rollinson D, Knopp S, Levitz S, Stothard JR, Tchuente LA, Garba A (2013). Time to set the agenda for schistosomiasis elimination. Acta Trop.

[3] Bergquist NR, Leonardo LR, Mitchell GF (2005). Vaccine-linked chemotherapy: can schistosomiasis control benefit from an integrated approach?. Trends Parasitol.

[4] McManus DP, Loukas A (2008). Current status of vaccines for schistosomiasis. Clin Microbiol Rev.

[5] Wilson RA, Coulson PS (2006). Schistosome vaccines: a critical appraisal. Mem Inst Oswaldo Cruz.

[6] Hotez PJ, Bethony JM, Diemert DJ, Pearson M, Loukas A (2010). Developing vaccines to combat hookworm infection and intestinal schistosomiasis. Nat Rev Microbiol.

[7] Tendler M, Simpson AJ (2008). The biotechnology-value chain: development of Sm14 as a schistosomiasis vaccine. Acta Trop.

[8] Brito CF, Oliveira GC, Oliveira SC, Street M, Riengrojpitak S, Wilson RA (2002). Sm14 gene expression in different stages of the *Schistosoma mansoni* life cycle and immunolocalization of the Sm14 protein within the adult worm. Braz J Med Biol Res.

[9] Almeida MS, Torloni H, Lee-Ho P, Vilar MM, Thaumaturgo N, Simpson AJ (2003). Vaccination against *Fasciola hepatica* infection using a *Schistosoma mansoni* defined recombinant antigen, Sm14. Parasite Immunol.

[10] Tendler M, Brito CA, Vilar MM, Serra-Freire N, Diogo CM, Almeida MS (1996). A *Schistosoma mansoni* fatty acid-binding protein, Sm14, is the potential basis of a dual-purpose anti-helminth vaccine. Proc Natl Acad Sci U S A.

[11] Cardoso FC, Macedo GC, Gava E, Kitten GT, Mati VL, de Melo AL (2008). *Schistosoma mansoni* tegument protein Sm29 is able to induce a Th1-type of immune response and protection against parasite infection. PLoS Negl Trop Dis.

[12] Correa-Oliveira R, Caldas IR, Gazzinelli G (2000). Natural versus drug-induced resistance in *Schistosoma mansoni* infection. Parasitol Today.

[13] Correa-Oliveira R, Pearce EJ, Oliveira GC, Golgher DB, Katz N, Bahia LG (1989). The human immune response to defined immunogens of *Schistosoma mansoni*: elevated antibody levels to paramyosin in stool-negative individuals from two endemic areas in Brazil. Trans R Soc Trop Med Hyg.

[14] Viana IR, Correa-Oliveira R, Carvalho Odos S, Massara CL, Colosimo E, Colley DG (1995). Comparison of antibody isotype responses to *Schistosoma mansoni* antigens by infected and putative resistant individuals living in an endemic area. Parasite Immunol.

[15] Viana IR, Sher A, Carvalho OS, Massara CL, Eloi-Santos SM, Pearce EJ (1994). Interferon-gamma production by peripheral blood mononuclear cells from residents of an area endemic for *Schistosoma mansoni*. Trans R Soc Trop Med Hyg.

[16] El Ridi R, Tallima H (2012). Adjuvant selection for vaccination against murine schistosomiasis. Scand J Immunol.

[17] Pinheiro CS, Ribeiro AP, Cardoso FC, Martins VP, Figueiredo BC, Assis NR (2014). A multivalent chimeric vaccine composed of Schistosoma mansoni SmTSP-2 and Sm29 was able to induce protection against infection in mice. Parasite Immunol.

[18] Alexopoulou L, Holt AC, Medzhitov R, Flavell RA (2001). Recognition of double-stranded RNA and activation of NF-kappaB by Toll-like receptor 3. Nature.

[19] Ewaisha RE, Bahey-El-Din M, Mossallam SF, Amer EI, Aboushleib HM, Khalil AM (2014). Combination of the two schistosomal antigens Sm14 and Sm29 elicits significant protection against experimental Schistosoma mansoni infection. Exp Parasitol.

[20] Cardoso FC, Pacifico RN, Mortara RA, Oliveira SC (2006). Human antibody responses of patients living in endemic areas for schistosomiasis to the tegumental protein Sm29 identified through genomic studies. Clin Exp Immunol.

[21] Chura-Chambi RM, Nakajima E, de Carvalho RR, Miyasato PA, Oliveira SC, Morganti L (2013). Refolding of the recombinant protein Sm29, a step toward the production of the vaccine candidate against schistosomiasis. J Biotechnol.

[22] Ribeiro F, Vieira Cdos S, Fernandes A, Araujo N, Katz N (2002). The effects of immunization with recombinant Sm14 (rSm14) in reducing worm burden and mortality of mice infected with Schistosoma mansoni. Rev Soc Bras Med Trop.

[23] Eissa MM, El-Azzouni MZ, Amer EI, Baddour NM (2011). Miltefosine, a promising novel agent for schistosomiasis mansoni. Int J Parasitol.

[24] Smithers SR, Terry RJ (1965). The infection of laboratory hosts with cercariae of Schistosoma mansoni and the recovery of the adult worms. Parasitology.

[25] Bahey-El-Din M, Casey PG, Griffin BT, Gahan CG (2008). Lactococcus lactis-expressing listeriolysin O (LLO) provides protection and specific CD8(+) T cells against Listeria monocytogenes in the murine infection model. Vaccine.

[26] Bahey-El-Din M, Griffin BT, Gahan CG (2008). Nisin inducible production of listeriolysin O in Lactococcus lactis NZ9000. Microb Cell Fact.

[27] Jefree CE, Read ND, Hall JL, Hawes CR (1991). Ambient- and Low-temperature scanning electron microscopy. Electron Microscopy of Plant Cells.

[28] Pritchard MH, Kruse GOW (1982). Stains and Staining Methods. The Collection and Preservation of Animal Parasites.

[29] Cheever AW (1968). Conditions affecting the accuracy of potassium hydroxide digestion techniques for counting Schistosoma mansoni eggs in tissues. Bull World Health Organ.

[30] Drury RAB, Wallington EA (1980). Carleton’s Histological Technique.

[31] Botros S, William S, Hammam O, Zidek Z, Holy A (2003). Activity of 9-(S)-[3-hydroxy-2-(phosphonomethoxy)propyl]adenine against Schistosomiasis mansoni in mice. Antimicrob Agents Chemother.

[32] Stryjewska A, Kiepura K, Librowski T, Lochynski S (2013). Biotechnology and genetic engineering in the new drug development. Part I. DNA technology and recombinant proteins. Pharmacol Rep.

[33] Fonseca CT, Brito CF, Alves JB, Oliveira SC (2004). IL-12 enhances protective immunity in mice engendered by immunization with recombinant 14 kDa *Schistosoma mansoni* fatty acid-binding protein through an IFN-gamma and TNF-alpha dependent pathway. Vaccine.

[34] Pacheco LG, Mati VL, Castro TL, Dorella FA, Oliveira SC, Miyoshi A (2008). Oral immunization with Salmonella harboring a Sm14-based DNA vaccine does not protect mice against Schistosoma mansoni infection. Parasitol Int.

[35] Ramos HR, Miyasato PA, Ramos CRR, de Mattos Arêas AP, Kawano T, Ho PL (2010). A Genetic Fusion between Sm14 and CTB does not Reduce Schistosoma mansoni Worm Burden on Intranasally Immunized BALB/c Mice. J Vaccines Vaccination.

[36] Martins VP, Pinheiro CS, Figueiredo BC, Assis NR, Morais SB, Caliari MV, et al. Vaccination with enzymatically cleaved GPI-anchored proteins from Schistosoma mansoni induces protection against challenge infection. Clin Dev Immunol 2012, 2012(in press). 10.1155/2012/962538 PMC3426240 22927873

[37] Visciano ML, Tagliamonte M, Tornesello ML, Buonaguro FM, Buonaguro L (2012). Effects of adjuvants on IgG subclasses elicited by virus-like particles. J Transl Med.

[38] Zouain CS, Gustavson S, Oliveira SC, Azevedo V, Alves JB, Goes AM (2001). The role of IL-10 and IgG1 in the protection and granulomatous response in Schistosoma mansoni P24-immunized mice. Vaccine.

[39] Al-Sherbiny M, Osman A, Barakat R, El Morshedy H, Bergquist R, Olds R (2003). In vitro cellular and humoral responses to Schistosoma mansoni vaccine candidate antigens. Acta Trop.

[40] Zhou R, Wei H, Tian Z (2007). NK3-like NK cells are involved in protective effect of polyinosinic-polycytidylic acid on type 1 diabetes in nonobese diabetic mice. J Immunol.

[41] Mian MF, Ahmed AN, Rad M, Babaian A, Bowdish D, Ashkar AA (2013). Length of dsRNA (poly I:C) drives distinct innate immune responses, depending on the cell type. J Leukoc Biol.

[42] Zhu X, Nishimura F, Sasaki K, Fujita M, Dusak JE, Eguchi J (2007). Toll like receptor-3 ligand poly-ICLC promotes the efficacy of peripheral vaccinations with tumor antigen-derived peptide epitopes in murine CNS tumor models. J Transl Med.

[43] Cheever AW, Lenzi JA, Lenzi HL, Andrade ZA (2002). Experimental models of Schistosoma mansoni infection. Mem Inst Oswaldo Cruz.

[44] Mati VL, Melo AL (2013). Current applications of oogram methodology in experimental schistosomiasis; fecundity of female Schistosoma mansoni and egg release in the intestine of AKR/J mice following immunomodulatory treatment with pentoxifylline. J Helminthol.

[45] Abdeen SH, Reda ES, El-Shabasy EA, Ouhtit A (2012). Ultrastructural changes of adult Schistosoma mansoni worms recovered from C57BL/6 mice passively immunized with normal and vaccinated rabbit sera in vivo. Parasitol Res.

[46] Gupta BC, Basch PF (1987). The role of Schistosoma mansoni males in feeding and development of female worms. J Parasitol.

[47] Basch PF (1990). Why do schistosomes have separate sexes?. Parasitol Today.

